# I will speak up if I feel energetic: Effects of supervisor humor on employee voice

**DOI:** 10.3389/fpsyg.2022.956499

**Published:** 2023-01-12

**Authors:** Daiheng Li, Pan Liu, Shuang Li, Jieya He

**Affiliations:** ^1^Business School, Beijing Wuzi University, Beijing, China; ^2^Beijing Institute of Petrochemical Technology, Beijing, China; ^3^Beijing Academy of Safety Engineering and Technology, Beijing, China; ^4^Northeastern University, Boston, MA, United States; ^5^Weifang Bureau of Commerce, Weifang, China

**Keywords:** supervisor humor, ego depletion, emotional intelligence, promotive voice, prohibitive voice

## Abstract

Extant literature on supervisor humor predominantly documents its beneficial effects on the organization, but its dark side receives little attention. Drawing on ego depletion theory, we proposed a conceptual model to examine the effects of two interpersonal types of supervisor humor (i.e., affiliative humor and aggressive humor) on employee voice. An empirical study with a sample covering 257 employees in China indicated that supervisor aggressive humor was negatively related to voice behaviors *via* depleting employees’ resource. In contrast, supervisor affiliative humor was positively related to voice behaviors owing to less depletion of employees. Contrary to prediction, emotional intelligence enhanced the positive effect of supervisor aggressive humor on employees’ depletion. Important theoretical and practical implications were discussed.

## Introduction

Recently, research on supervisor humor in the organization has received increasing interest among scholars (Gkorezis et al., 2011; Huo et al., 2012; Pundt and Herrmann, 2015; Kim et al., 2016; Cooper et al., 2018; Mesmer-Magnus et al., 2018; Liu et al., 2019; Tan et al., 2020; Rosenberg et al., 2021). Supervisor humor, a behavior enacted directed toward a subordinate by a supervisor that is intended to be amusing to the subordinate and that the subordinate perceives the act as intentional (Cooper, 2005; Cooper et al., 2018), is particularly salient since supervisors hold power and have control over resources, and thus set the tone for humor use in the organization (Cooper, 2005; Pundt and Herrmann, 2015; Li et al., 2021; Wu et al., 2021; Chen et al., 2022). Empirical research has indicated that supervisor humor is positively related to task performance (Avolio et al., 1999), organizational citizenship behaviors (Cooper et al., 2018), innovative behavior (Pundt, 2015), work engagement (Yam et al., 2018), job satisfaction (Mesmer-Magnus et al., 2018), and leader-member exchange (Pundt and Herrmann, 2015; Tremblay, 2021).

Despite those promising findings, several critical issues about supervisor humor remain unresolved. First, recent supervisor humor studies have focused on relational processes or employees’ in-role behaviors (Kim et al., 2016; Pundt and Venz, 2017; Yam et al., 2018), but have ignored the extra-role behavior in the form of voice behavior defined as a discretionary behavior that expresses constructive ideas and suggestions intended to improve the organization (Van Dyne and LePine, 1998; Burris et al., 2017). Practically, the effects of supervisor humor on employee voice behavior should be taken seriously, considering the critical role of employee voice behavior in improving organizational performance, identifying issues, and preventing failure (Liang et al., 2012; Lin and Johnson, 2015; Weiss and Morrison, 2019; Liu, 2022). Hence, examining the relationship between supervisor humor and employee voice is critically significant. Second, there is a shortage of research into the effects of supervisor humor on voice behaviors. In the prior study, some scholars have illustrated the roles of LMX and burnout in the relationships between supervisor humor and voice behaviors, whereas they have ignored the role of employees’ psychological resources (Cooper et al., 2018; Liu et al., 2019). Additionally, employees are heterogeneous in terms of their characteristics. Notwithstanding, the role of personal characteristics in the relationship between supervisor humor and its potential consequences has not been sufficiently explored (Cooper et al., 2018; Mesmer-Magnus et al., 2018).

**Figure 1:**
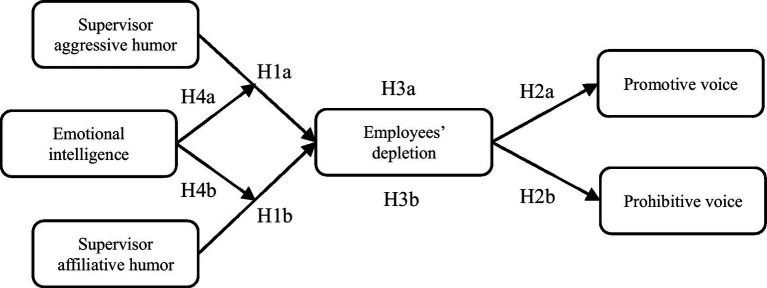
Conceptual model.

To address these issues, our study aims to investigate the effects of two interpersonal types of supervisor humor – supervisor affiliative humor and supervisor aggressive humor (Martin et al., 2003; Pundt and Herrmann, 2015) – on employee voice. To shed light on this process, we adopt a psychological resource perspective to explore how supervisor humor affects employees’ voice behaviors based on ego depletion theory. According to ego depletion theory (Baumeister et al., 1998), regulating behaviors can deplete individuals’ limited resources. Nevertheless, not all behaviors are the same regarding the depletion they cause (Lin and Johnson, 2015). Indeed, we predict that supervisor affiliative humor and supervisor aggressive humor have opposing effects on depletion, influencing employees’ subsequent voice behavior. Furthermore, we select promotive voice, which involves expressing new ideas and suggestions to improve organizational functioning, and prohibitive voice, which involves expressing concerns and worries to prevent organizational failure that has been generally recognized by scholars as the outcome (Liang et al., 2012; Huang et al., 2018), to illustrate whether supervisor humor exerts the same influence on different types of voice behavior (Tan et al., 2020). In addition, personal characteristics should be considered to understand the boundary conditions under which supervisor humor affects employee voice (Cooper et al., 2018). One of the critical factors is emotional intelligence (EI), defined as individuals’ capability to identify and regulate emotions in themselves and others (Salovey and Mayer, 1990). Some scholars have proven that emotional intelligence significantly affects how individuals respond to others’ behaviors (Law et al., 2004; Carmeli and Josman, 2006; Miao et al., 2020). Accordingly, the effects of supervisor humor on employee voice behavior may vary according to the level of emotional intelligence. Our proposed model is illustrated in Figure 1.

Our research makes several theoretical contributions to the existing literature. First, we contribute to the research on humor by elaborating whether, how, and under what conditions supervisor humor affects employee voice. In doing so, this study enriches humor literature by exploring its downstream effects on employee voice. Second, our study advances the voice literature by examining supervisor humor as a critical antecedent. This study offers a timely response to the recent call for exploring the correlation between humor and voice (Morrison, 2011; Tan et al., 2020). Third, this study extends the ego depletion theory by revealing the mediating role of ego depletion. By adopting a psychological resources perspective, this study provides a new theoretical view and explanation of how supervisor humor affects employee voice.

## Theory and hypotheses

### Supervisor humor and employees’ depletion

Grounded in ego depletion theory (Baumeister et al., 1998), self-regulatory resources are critically important because they are required by all self-regulation activities. Once depleted, individuals will succumb to aggressive impulses and addictive behaviors (Christian and Ellis, 2011; Barnes et al., 2015). Hence, identifying potential sources of resource depletion and replenishment is an essential first step to help employees regulate their behaviors (Beal et al., 2005). Individuals’ self-control resources at work are significantly affected by daily interpersonal events (Lilius, 2012; Bono et al., 2013), which we suspect supervisor aggressive humor and supervisor affiliative humor are included.

Supervisor aggressive humor, defined as a negative form of humor to denigrate, disparage, excessively tease, or ridicule others (Martin et al., 2003), may deplete employees’ self-regulatory resources. First, previous research has illustrated that stress or strain is one of the critical factors affecting depletion (Baumeister et al., 2005; Oaten and Cheng, 2006). Supervisor aggressive humor may make employees caught in intimidation and embarrassment, gradually developing a stressor for employees (Kim et al., 2016). In this instance, employees have to consume limited resources to deal with stress, which in turn increases depletion (Liu et al., 2021). Second, supervisor aggressive humor coincides with negative emotions (Huo et al., 2012; Goswami et al., 2015), which are more depleting relative to positive emotions. Employees ridiculed by supervisors suffer anxiety and agitation. In the workplace, employees have to regulate their emotions, especially negative ones (Grandey, 2003), yet doing so is depleting because energy is required to align their emotional states with company display rules and norms (Muraven and Baumeister, 2000). Moreover, employees usually cannot take “revenge” when they suffer aggressive humor from their supervisors because they still count on their supervisors (Huo et al., 2012). Thus, employees must not only control their own negative emotions but mend or maintain relational ties with their supervisors, resulting in their depletion (Muraven, 2012). Hence, we predict:

*Hypothesis 1a*: Supervisor aggressive humor is positively related to employees’ depletion.

Supervisor affiliative humor, a positive form of humor to amuse others (Martin et al., 2003), may result in less subsequent depletion. First, employees may feel more comfortable at work because supervisor affiliative humor conveys support, amicability, and trust (Pundt and Herrmann, 2015; Cooper et al., 2018). When employees feel relaxed, they may be less depletion because they require less self-control and restraint (Ryan and Deci, 2008). Second, experiencing positive emotions has been proven to counteract depletion (Tice et al., 2007). When supervisors interact with their subordinates through funny stories, jokes, or spontaneous witty banter, they can create a warm working environment and elicit positive emotions *via* emotional contagion. Accordingly, employees who experience positive emotions can have remarkable power to restore their capacity and willingness to exert control and volition (Mesmer-Magnus et al., 2018). Third, ego depletion theory assumes that regulatory resources can be bolstered when individuals experience positive social interactions (Baumeister et al., 2007; Bono et al., 2013). Supervisor affiliative humor is a form of self-disclosure, which is conducive to close relationships between supervisors and employees (Cooper, 2008). An intimate relationship with supervisors can help employees increase psychological safety and reduce uncertainty (Mesmer-Magnus et al., 2018), which in turn replenishes regulatory resources. Hence, we propose:

*Hypothesis 1b*: Supervisor affiliative humor is negatively related to employees’ depletion.

### Employees’ depletion and voice behaviors

Drawing on ego depletion theory, depletion dampens individuals’ self-control on subsequent tasks (Baumeister et al., 1998). Empirical research has illustrated that depletion can reduce individuals’ capacity to solve complex problems (Schmeichel et al., 2003), engage in impression management (Vohs et al., 2005), deal with demanding partners (Muraven, 2012), inhibit deviant behaviors and unethical impulses (Thau and Mitchell, 2010; Barnes et al., 2011; Christian and Ellis, 2011), suppress aggressive responses (Stucke and Baumeister, 2006), and engage in constructive work activities (Johnson et al., 2014; Lanaj et al., 2014). Consequently, employees are not likely to have the necessary resources to enact voice behavior regardless of whether it is promotive or prohibitive voice when they feel depleted. Employee voice is a discretionary and extra-role behavior that requires employees to invest effort and energy (Van Dyne and LePine, 1998). To conserve energy and accomplish in-role duties, depleted employees have to scale back prosocial discretionary behaviors (DeWall et al., 2008; Johnson et al., 2014).

Moreover, depletion can decrease rather than increase risky behaviors (Unger and Stahlberg, 2011). Employee voice is potentially risky because it aims to challenge the status quo (Fast et al., 2014; Dalal and Sheng, 2018). Although promotive voice involves expressing ideas and suggestions for moving toward ideal states, others may dislike the suggestion or see it as disruptive (Fast et al., 2014; Dalal and Sheng, 2018). Also, prohibitive voice may increase task conflicts with colleagues because it often points out problems with others’ work activities (Burris, 2012; Liang et al., 2012). When employees confront the potential dangers or negative consequences of a risky decision, they have to deal with regret and negative feelings immediately (Unger and Stahlberg, 2011). As a result, employees may refrain from voice behaviors because they fear that they have not enough resources to deal with the adverse outcomes. Taken together, we propose:

*Hypothesis 2*: employees’ depletion is negatively related to (a) promotive voice and (b) prohibitive voice.

Ego depletion theory proposes that depletion caused by initial exertions of self-control dampens individuals’ self-control on subsequent tasks (Baumeister et al., 1998). Supervisor humor as a form of interpersonal event can significantly affect individuals’ self-control resources (Lilius, 2012; Muraven, 2012; Bono et al., 2013).

Supervisor aggressive humor signals offense and humiliation (Martin et al., 2003; Pundt and Herrmann, 2015), which causes employees to strain and feel anxious. In this instance, employees require to invest resources to cope with impulses and negative emotions. Naturally, this process results in resource loss and then increases ego depletion, prohibiting employees from speaking up. In contrast, supervisor affiliative humor aims to amuse others and make them feel comfortable (Martin et al., 2003; Pundt and Herrmann, 2015), which is beneficial for creating a positive atmosphere in social interactions (Kuiper and Leite, 2010). Employees may be less depleted and have more resources to exert subsequent voice behaviors when experiencing relaxing and positive social interactions. Based on the preceding discussion, we propose:

*Hypothesis 3a*: Employees’ depletion mediates the negative effect of supervisor aggressive humor on voice behaviors.

*Hypotheses 3b*: Employees’ depletion mediates the positive effect of supervisor affiliative humor on voice behaviors.

### The moderating role of emotional intelligence

In the preceding section, we proposed that supervisor aggressive humor depletes regulatory resources, whereas depletion is less when employees experience affiliative humor from supervisor. In this section, we discuss an individual difference variable – emotional intelligence – that, according to ego depletion theory, influences how individuals experience a change in their regulatory resources (Hagger et al., 2010). Emotional intelligence refers to the ability to perceive, regulate, and manage emotions to promote emotional and intellectual growth (Salovey and Mayer, 1990; Davies et al., 1998; Grover and Furnham, 2020). Emotionally intelligent individuals are well at perceiving and managing others’ emotions. Previous research has indicated that emotional intelligence is related to psychological well-being (Carmeli et al., 2009), task performance (Joseph and Newman, 2010), positive moods, and higher self-esteem (Schutte et al., 2002).

According to ego depletion theory, emotional intelligence may diminish the effect of depletion (Tice et al., 2007; Hagger et al., 2010; Johnson et al., 2014). Confronted with supervisor affiliative humor, employees with high emotional intelligence may be more likely to perceive the supervisor’s goodwill and experience positive emotions, which is beneficial for conserving regulatory resources. Thus, emotional intelligence guides employees to reduce the need for self-control and even replenish resources. Similarly, emotional intelligence may buffer against the negative effect of supervisor aggressive humor. Research has indicated that emotionally intelligent employees can maintain positive mental states due to their ability to effectively manage their emotions (Salovey and Mayer, 1990). By this logic, emotionally intelligent employees can handle aggression or offense better than others; even if they experience anxiety or stress from supervisor aggressive humor, they can cope better (Grover and Furnham, 2020). Accordingly, emotionally intelligent employees are likely to experience a lower level of depletion when suffering tease or ridicule from supervisors. For the reasons above, we propose:

*Hypothesis 4a*: emotional intelligence weakens the positive effect of supervisor aggressive humor on employees’ depletion.

*Hypothesis 4b*: emotional intelligence enhances the negative effects of supervisor affiliative humor on employees’ depletion.

## Materials and methods

### Sample and procedure

An online survey was executed to gather data from 346 full-time employees in a finance company in Northern China. After acquiring the permission of managers, we described the survey for employees by Voov Meeting. We explained to all employees and guaranteed that the survey was voluntary, confidential, anonymous, and irrelevant to their performance evaluation. Then, employees who agreed to participate in the survey were directed to the WeChat Group. With a list of names from HR, codes were assigned to each participant. Measures of the different variables were randomized across participants to control for order bias (Dillman et al., 2014). To minimize potential common method biases and reduce participants’ fatigue (Podsakoff et al., 2003, 2012), we used a three-wave method for the data collection, with each wave separated by 1 month. In time 1, we collected demographic variables, supervisor humor, and emotional intelligence. In time 2, employees’ depletion was evaluated. In time 3, promotive voice and prohibitive voice were measured.

The final sample comprised 257 valid questionnaires, with an overall response rate of 74.28%. Of the 257 participants, 174 (69.1%) were women, and 83 (30.9%) were men. There were 4 (1.6%) who were postgraduates, 153 (59.5%) who were undergraduates, and 100 (38.9%) who had graduated from junior college. They ranged in age from 18 to 30 years (15.2%), 31–40 years (43.6%), and 41 years and older (41.2%). 26.1% of participants had worked for less than 1 year, 32.7% for 1–3 years, and 41.2% for 4 years and more.

### Measures

To ensure the validity and appropriateness of the measures in the Chinese context, a Chinese version of all measures was developed following a standard translation and back-translation procedure suggested by Brislin (1986). For all measures, we used a five-point Likert-type scale ranging from 1 (completely disagree) to 5 (completely agree).

#### Supervisor aggressive humor

We adopted an 8-item scale (Cronbach’s *α* = 0.881) developed by Martin et al. (2003). This scale includes the following sample item: “If my supervisor does not like someone, he/she often uses humor or teasing to put them down.”

#### Supervisor affiliative humor

We used an 8-item scale (Cronbach’s *α* = 0.890) developed by Martin et al. (2003). A sample item was “My supervisor enjoys making people laugh.”

#### Depletion

According to Lin and Johnson (2015), we adopted a 5-item scale (Cronbach’s *α* = 0.904) developed by Twenge et al. (2004). Sample items include: “I feel drained” and “My mind feels unfocused right now.”

#### Voice behavior

Voice behaviors were measured using items developed by Liang et al. (2012). Promotive voice (Cronbach’s *α* = 0.945) and prohibitive voice (Cronbach’s *α* = 0.836) were each assessed *via* five items. A sample item of promotive voice includes: “I proactively suggest new projects which are beneficial to the work unit..” A sample item of prohibitive voice includes: “I proactively report coordination problems in the workplace to the management.”

#### Emotional intelligence

We used a 16-item scale (Cronbach’s *α* = 0.969) developed by Law et al. (2004) to assess emotional intelligence. Sample items include: “I have a good understanding of my own emotions.” and “I am a good observer of others’ emotions.”

#### Control variables

We controlled for an assortment of variables, including age, gender, education, and tenure.

### Analytic strategy

First, confirmatory factor analyses (CFA) were conducted by using Mplus 8.0 (Muthén and Muthén, 2012) to examine the validity of the measures (Hooper et al., 2008). Second, we conducted path analysis and bootstrapping approach (Preacher and Hayes, 2008; Zhao and Chen, 2010) to test for the direct and indirect effect of supervisor humor in SPSS 25.0. Finally, we examined the hypothesized mediation model by incorporating emotional intelligence into the model and calculated the conditional effects with bias-corrected confidence intervals (Hayes, 2013).

## Results

### Confirmatory factor analysis

Confirmatory factor analysis (CFA) was executed with Mplus 8.0 to examine the validity of six key constructs. First, a six-factor CFA model, including supervisor aggressive humor, supervisor affiliative humor, employees’ depletion, emotional intelligence, promotive voice, and prohibitive voice, was examined. As shown in Table 1, the results revealed a good fit for the theorized six-factor model (*χ ^2^* (725) =1428.436, CFI = 0.910, TLI = 0.903, RMSEA = 0.061, SRMR = 0.046). Several comparisons with alternative models were made to confirm that the six-factor model was the best structure to apply. The results in Table 1 showed that the six-factor model fitted the data better than any of the competing models. The validity of our specified measurement model was supported.

**Table 1:** Results of confirmatory factor analysis.

**Model**	***χ***^***2***^	***df***	***χ***^***2***^***/df***	**CFI**	***TLI***	***RMSEA***	***SRMR***
Six-factor	1428.436	725	1.970	0.910	0.903	0.061	0.046
Five-factor: ED + EI	1755.389	730	2.405	0.869	0.860	0.074	0.056
Four-factor: SAGH+SAFH, ED + EI	1807.455	734	2.462	0.863	0.855	0.075	0.059
Four-factor: SAGH+SAFH, PMV + PHV	1502.119	734	2.046	0.902	0.896	0.064	0.052
Four-factor: ED + EI, PMV + PHV	1770.085	734	2.412	0.868	0.860	0.074	0.057
Three-factor: SAGH+SAFH, ED + EI, PMV + PHV	1821.138	737	2.471	0.862	0.854	0.076	0.060
Two-factor: SAGH+SAFH+PMV + PHV, ED + EI	3246.399	739	4.393	0.680	0.662	0.115	0.101
One-factor	3943.818	740	5.329	0.591	0.569	0.130	0.114

ED, employees’ depletion; EI, emotional intelligence; SAGH, supervisor aggressive humor; SAFH, supervisor affiliative humor; PMV, promotive voice; PHV, prohibitive voice.

### Descriptive analyses

Means, standard deviations, reliabilities, and zero-order correlations of variables are shown in Table 2. Supervisor affiliative humor is negatively related to employees’ depletion (*r* = −0.532, *p* < 0.01), positively associated with promotive voice (*r* = 0.400, *p* < 0.01) and prohibitive voice (*r* = 0.304, *p* < 0.01). Supervisor aggressive humor is positively related to employees’ depletion (*r* = 0.642, *p* < 0.01), negatively associated with promotive voice (*r* = −0.390, *p* < 0.01) and prohibitive voice (*r* = −0.342, *p* < 0.01). Moreover, employees’ depletion is negatively related to promotive voice (*r* = −0.471, *p* < 0.01) and prohibitive voice (*r* = −0.377, *p* < 0.01).

**Table 2:** Means, standard deviations, reliabilities, and correlations.

**Variables**	**1**	**2**	**3**	**4**	**5**	**6**	**7**	**8**	**9**	**10**
Gender	1									
Age	0.021	1								
Education	−0.148^*^	−0.438^**^	1							
Tenure	−0.042	0.173^**^	0.226^**^	1						
Supervisor affiliative humor	0.061	−0.046	−0.070	−0.075	***0.890***					
Supervisor aggressive humor	−0.152^*^	0.042	0.063	0.075	−0.792^**^	***0.881***				
Employees’ depletion	−0.046	0.061	−0.094	−0.006	−0.532^**^	0.642^**^	***0.904***			
Emotional intelligence	−0.016	0.007	0.079	0.081	0.499^**^	−0.558^**^	−0.702^**^	***0.969***		
Promotive voice	0.001	0.044	0.031	0.044	0.400^**^	−0.390^**^	−0.471^**^	0.543^**^	***0.945***	
Prohibitive voice	−0.010	0.117	0.007	0.004	0.304^**^	−0.342^**^	−0.377^**^	0.435^**^	0.739^**^	***0.836***
Mean	0.677	3.315	2.459	2.447	3.660	2.368	2.307	3.800	3.926	3.665
*SD*	0.469	0.865	0.785	1.169	0.732	0.695	0.887	0.720	0.702	0.672

N = 257. Cronbach’s α values are shown along the diagonal in bold italics.

^*^p < 0.05. ^**^p < 0.01.

### Test of hypotheses

**Figure 2:**
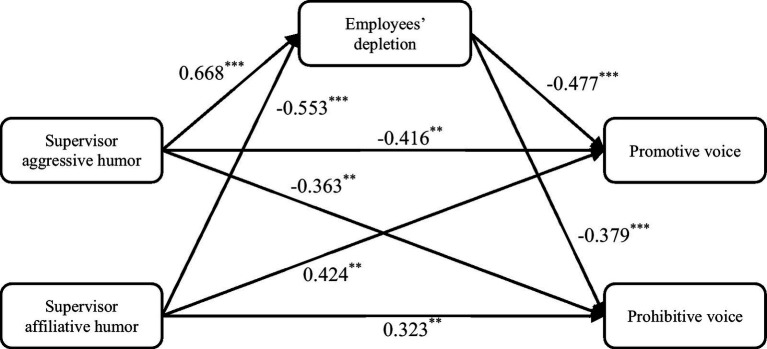
Results of path analysis. N = 257. **p < 0.01. ***p < 0.001.

Path analysis was utilized to test hypotheses 1a, 1b, 2a, and 2b. As summarized in Figure 2, the positive effect of supervisor aggressive humor on employees’ depletion was significant after including the controls (*β* = 0.668 *p* < 0.001), and the negative effect of supervisor affiliative humor on employees’ depletion was also significant (*β* = −0.553, *p* < 0.001). In addition, the significantly negative effects of employees’ depletion on promotive voice (*β* = −0.477, *p* < 0.001) and prohibitive voice (*β* = −0.379, *p* < 0.001) were verified. Consequently, H1a, 1b, 2a, and 2b were supported.

All remaining hypotheses were tested using the PROCESS macro in SPSS 25.0 (Hayes, 2013) with a 5,000-resample bootstrap method (Preacher et al., 2007). To examine hypothesis 3a, PROCESS model 4 was executed. As shown in Table 3, the result illustrated the significantly indirect effect of employees’ depletion on the “supervisor aggressive humor – promotive voice” relationship (*E.S.* = −0.244, 95% bias-corrected *CI* = [−0.417, −0.118]) as well as “supervisor aggressive humor – prohibitive voice” relationship (*E.S.* = −0.165, 95% bias-corrected *CI* = [−0.307, −0.060]). Thus, H3a was supported.

**Table 3:** Employees’ depletion as a mediator in the relationship between supervisor humor and voice behaviors.

**Independent variables**	**Dependent variables**	**Effect**		**Boot SE**	**Boot LL 95% CI**	**Boot UL 95% CI**
Supervisor aggressive humor	Promotive voice	Direct effect	−0.169	0.119	−0.403	0.066
		Indirect effect	−0.244	0.078	−0.417	−0.118
	Prohibitive voice	Direct effect	−0.185	0.099	−0.379	0.009
		Indirect effect	−0.165	0.062	−0.307	−0.060
Supervisor affiliative humor	Promotive voice	Direct effect	0.213	0.093	0.03	0.395
		Indirect effect	0.185	0.052	0.095	0.301
	Prohibitive voice	Direct effect	0.145	0.082	−0.017	0.307
		Indirect effect	0.148	0.049	0.064	0.253

All coefficients are unstandardized. SE, standard error; LL, lower level; UL, upper level.

Likewise, PROCESS model 4 was applied to test hypothesis 3b. The result, as shown in Table 3, revealed the significant indirect effect of supervisor affiliative humor on promotive voice (*E.S.* = 0.185, 95% bias-corrected *CI* = [0.095, 0.301]) and prohibitive voice (*E.S.* = 0.148, 95% bias-corrected *CI* = [0.064, 0.253]). Hence, H3b was supported.

**Figure 3:**
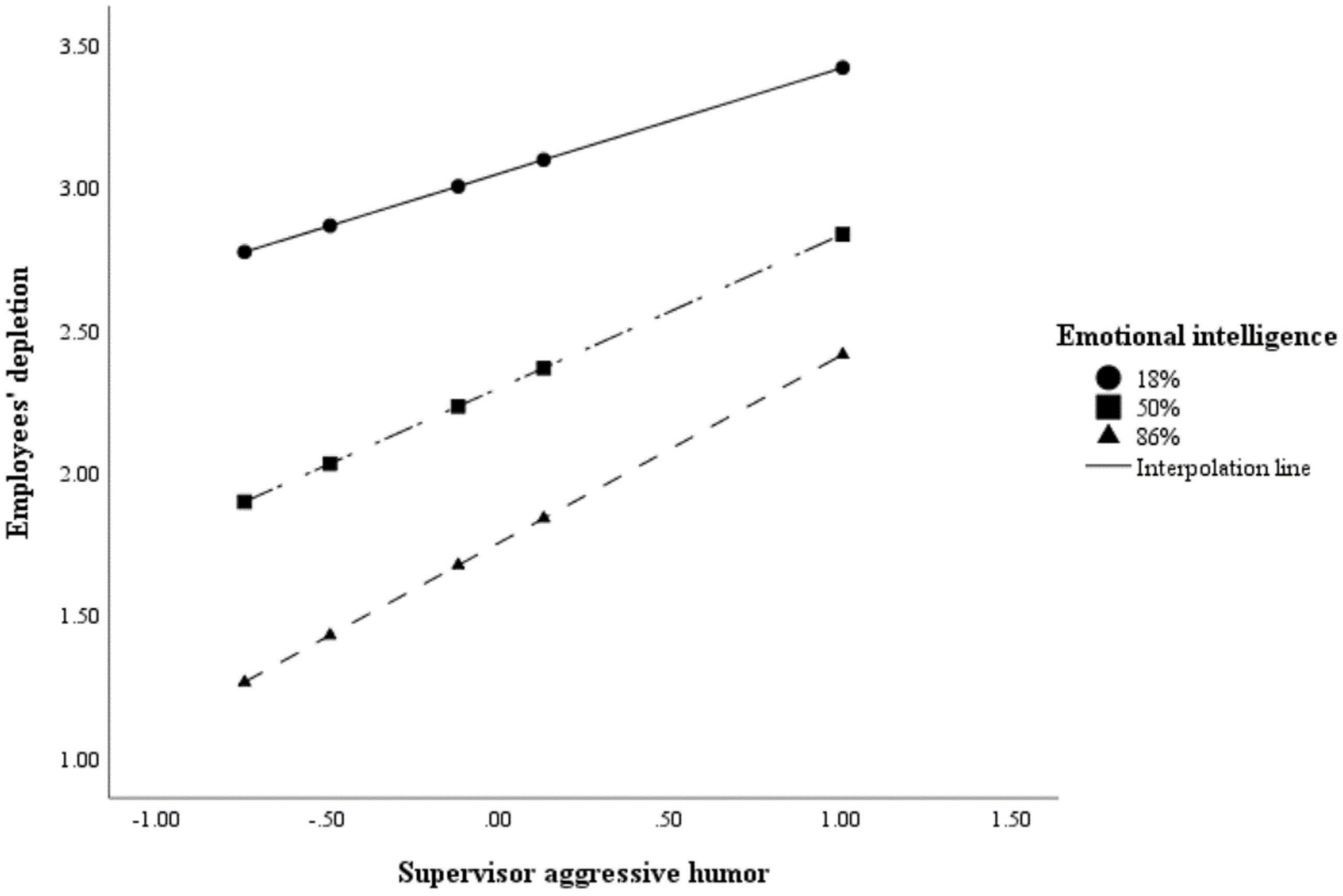
Interactive effect of supervisor aggressive humor and emotional intelligence on employees’ depletion.

PROCESS model 1 was executed to test H4a. As shown in Table 4, it revealed that the interaction between supervisor aggressive humor and emotional intelligence was significantly related to employees’ depletion (*E.S.* = 0.148, *SE* = 0.059, 95% bias-corrected *CI* = [0.031, 0.265]). Following Hayes (2013), we plotted the interactions at 18, 50, and 86% percentiles of emotional intelligence. As shown in Figure 3, the effect of supervisor aggressive humor on employees’ depletion was stronger for emotionally intelligent employees. Nevertheless, this result was contrary to expectations. Thus, H4a was not supported.

**Table 4:** Emotional intelligence as a moderator in the relationship between supervisor aggressive humor and employees’ depletion.

**Variables**	**Effect**	**SE**	**Boot LL 95% CI**	**Boot UL 95% CI**
Y: Employees’ depletion				
Constant	2.556	0.252	2.060	3.053
M: Emotional intelligence	−0.668	0.075	−0.817	−0.519
X: Supervisor aggressive humor	0.524	0.095	0.338	0.711
Interaction: X × M	0.148	0.059	0.031	0.265

All coefficients are unstandardized. SE, standard error; LL, lower level; UL, upper level.

Again, PROCESS model 1 was applied to test H4b. As shown in Table 5, the result illustrated that the interaction between supervisor affiliative humor and emotional intelligence was not significantly related to employees’ depletion (*E.S.* = −0.037, *SE* = 0.057, 95% bias-corrected *CI* = [−0.149, 0.075]). Hence, H4b was not supported.

**Table 5:** Emotional intelligence as a moderator in the relationship between supervisor affiliative humor and employees’ depletion.

**Variables**	**Effect**	**SE**	**Boot LL 95% CI**	**Boot UL 95% CI**
Y: Employees’ depletion				
Constant	2.472	0.265	1.949	2.995
M: Emotional intelligence	−0.728	0.080	−0.886	−0.571
X: Supervisor affiliative humor	−0.295	0.077	−0.447	−0.144
Interaction: X × M	−0.037	0.057	−0.149	0.075

All coefficients are unstandardized. SE, standard error; LL, lower level; UL, upper level.

In addition, PROCESS model 7 was applied to test the conditional effects of EI on the mediation chain. As shown in Table 6, the indirect effect of supervisor aggressive humor on promotive voice and prohibitive voice *via* employees’ depletion was stronger when emotional intelligence was high. The results indicated that emotional intelligence enhanced the indirect effect of supervisor aggressive humor on voice behaviors *via* employees’ depletion. As shown in Table 7, the indirect effect of supervisor affiliative humor on promotive voice and prohibitive voice *via* employees’ depletion was stronger when emotional intelligence was high. The results suggested that emotional intelligence enhanced the indirect effect of supervisor affiliative humor on voice behaviors *via* employees’ depletion.

**Table 6:** Results of the moderated path analysis (a).

Dependent variables	Emotional intelligence	Supervisor aggressive humor → employees’ depletion → voice behaviors			
		Effect	Boot SE	Boot LL95%CI	Boot LL95 CI
Promotive voice	Low	−0.121	0.051	−0.248	−0.046
	High	−0.183	0.062	−0.331	−0.087
Prohibitive voice	Low	−0.082	0.040	−0.182	−0.025
	High	−0.124	0.050	−0.243	−0.045

**Table 7:** Results of the moderated path analysis (b).

Dependent variables	Emotional intelligence	Supervisor affiliative humor → employees’ depletion → voice behaviors			
		Effect	Boot SE	Boot LL95%CI	Boot LL95 CI
Promotive voice	Low	0.075	0.037	0.019	0.164
	High	0.089	0.031	0.041	0.164
Prohibitive voice	Low	0.060	0.032	0.015	0.144
	High	0.072	0.026	0.030	0.135

## Discussion

Grounded in ego depletion theory, this study proposes and verifies the model to explore the mechanism by which two interpersonal aspect of supervisor humor (i.e., aggressive humor and affiliative humor) affects employees’ voice. Results indicate that supervisor aggressive humor is negatively related to voice behaviors by depleting employees’ resources. In contrast, supervisor affiliative humor is positively related to voice behaviors owing to less depletion of employees. Contrary to expectations, emotionally intelligent employees are more likely to deplete resources when they experience aggressive humor from the supervisor. According to ego depletion theory, regulating emotion leads to the depletion of self-control resources (Baumeister et al., 1998). Emotionally intelligent employees are more sensitive to others’ emotions, so they may sharply perceive the aggression or offense behind supervisor aggressive humor, which causes them to feel anxious and stressed (Law et al., 2004). To mitigate negative reactions, emotionally intelligent employees must consume more resources to control their negative emotional states. Thus, emotional intelligence can amplify the effects of supervisor aggressive humor.

In addition, the moderating effect of emotional intelligence on the relationship between supervisor affiliative humor and employees’ depletion is insignificant. As a socioemotional resource, supervisor affiliative humor can inoculate employees from stress and burnout or help subordinates bounce back from stress (Cooper et al., 2018) rather than depleting them. At the same time, emotional intelligence can help employees to manage stress (Grover and Furnham, 2020). Thus, supervisor affiliative humor does not consume the resources of employees, whether they are emotionally intelligent or not.

### Theoretical implications

In examining those hypotheses, the findings of our study make several critical theoretical implications for the research. First, our study extends research on supervisor humor by explicating resource depletion as a key mechanism by which supervisor humor affects employee behaviors. Past research on supervisor humor has underlined the meaning of relational processes (Cooper et al., 2018). There is, however, a lack of attention to the psychological resource states of humor targets. Grounded in ego depletion theory, this study finds that supervisor humor exerts significant effects on voice behavior *via* consuming or replenishing employees’ resources. Ego depletion serves as a bridge linking supervisor humor to employee voice. Our study responds to the call from Yam et al. (2018) that reveals the potential mediating mechanisms in which supervisor humor affects employee behaviors.

Second, our study clarifies whether supervisor humor exerts the same influence on the different types of voice behavior. Voice behavior is classified as promotive or prohibitive based on whether employee voice is expressing suggestions or concerns (Liang et al., 2012). Does supervisor humor exert the same influence on promotive and prohibitive voices? This is an interesting research issue. However, prior research has neglected this (Liu et al., 2019; Tan et al., 2020). In our study, the empirical results demonstrate that supervisor humor exerts the same influence on different types of voice behavior. Specifically, supervisor aggressive humor is negatively related to promotive and prohibitive voice, while supervisor affiliative humor is positively related to promotive and prohibitive voice. Accordingly, our study illustrates that considering different voice styles is unnecessary when examining the relationships between supervisor humor and employee voice.

Moreover, in response to the call from Mesmer-Magnus et al. (2018), the important implication of our study is the investigation of the boundary conditions of the effects of supervisor humor in the organization. Although the result is contrary to our prediction and does not align with previous research that has illustrated emotional intelligence can buffer the negative effects of interpersonal conflict (Grover and Furnham, 2020), these findings still extend prior studies and indicate that emotional intelligence moderates the effects of supervisor aggressive humor on employees’ depletion. These results are vital for developing and refining humor theory about how individual differences moderate the effects of supervisor humor, though additional research is required on this issue.

### Practical implications

There are several important implications for managerial practices in our research. First, humor as an effective management tool should be recommended in the organization. Research has indicated that supervisor humor is always associated with improving performance and increasing job satisfaction and self-esteem (Gkorezis et al., 2011; Kim et al., 2016; Mesmer-Magnus et al., 2018). Meanwhile, supervisors should note that humor can impede employee behaviors. Our results indicated that supervisor affiliative humor was beneficial for employee voice, whereas supervisor aggressive humor impeded employee voice. Although aggressive humor from supervisor may not be intentional, it also can cause employees to fall into anxiety and strain. Hence, organizations should provide training to guide supervisors in understanding appropriate ways to use humor; a funny joke is a medicine, but sarcasm is poison.

Second, it has been proved that depletion is positively related to deviant behaviors (Thau and Mitchell, 2010) and aggressive behaviors (Stucke and Baumeister, 2006) but negatively associated with helping behaviors (DeWall et al., 2008). Our findings illustrated that supervisor aggressive humor could cause employees’ depletion while supervisor affiliative humor may replenish employees’ regulatory resources. Accordingly, supervisors should focus on the psychological resource state of employees. For example, supervisors can tell a funny joke to eliminate the negative impact caused by depletion when employees fall into anxiety or confusion. Additionally, supervisors can provide more rest time and increase organizational commitment to activate employees’ positive emotions, which in turn helps them replenish resources.

Finally, emotional intelligence is a critical moderator of the association between supervisor humor and employees’ depletion. The result indicated that emotionally intelligent employees were more vulnerable to depletion when mocked or ridiculed by their supervisors because they were more sensitive to malignant information behind sarcasm (Grover and Furnham, 2020). Hence, these employees should learn how to manage their emotions and moods, and try to decrease excessive resources for interpersonal relationships. It is more likely to protect ourselves by delaying an instant response. In this way, employees have enough time and energy to deal with subsequent negative emotions or moods and present the desired image (Vohs et al., 2005).

### Limitations and future research

The limitations in our study indicate several possible directions for future research. First, this study only concentrated on the interpersonal aspect of supervisor humor and neglected self-directed humor styles (i.e., self-enhancing humor and self-defeating humor; Martin et al., 2003). This study indicated that the interpersonal aspect of supervisor humor could directly affect employees’ psychological resources and further influence their behaviors. Self-directed humor is also likely related to employees’ depletion and behaviors directly or indirectly. For example, self-enhancing humor, defined as a generally humorous outlook on life (Martin et al., 2003), may be essential for supervisors to maintain positive attitudes and behaviors even in stressful situations. Through emotional contagion (Barsade, 2002), employees may be inspired to regulate negative emotions and deal with adverse situations actively.

Second, although the results of CFA (Table 1) revealed a good fit for the theorized six-factor model, common method variance could still be a concern because we collected data from the same source. Several procedural remedies were executed to reduce potential bias (Podsakoff et al., 2003): First, all participants were guaranteed that the survey was voluntary, confidential, anonymous, and irrelevant to their performance evaluation to reduce their evaluation apprehension or social desirability biases. Second, different instructions were adopted to construct psychological separation in the survey. In this way, participants were unlikely to perceive direct relations between the variables. Nevertheless, we encourage future research to replicate the results based on different sources (i.e., employees, peers, and supervisors) through a multi-wave research design.

Third, we only investigated employees’ depletion as mediators of the effects of supervisor humor. According to the extant literature, there is a range of alternative processes affected by supervisor humor, such as work engagement, LMX, or psychological safety, which might as well affect voice behaviors (Kim et al., 2016; Pundt and Venz, 2017; Yam et al., 2018). Nevertheless, we failed to test these alternative mechanisms. Hence, we encourage future research to test alternative meditation paths to illustrate that ego depletion is a potential mechanism with additive value.

Finally, cultural differences or specifics may more or less affect our results. Prior research has indicated individuals tend to make them spend more time and strength on relationships with colleagues in China, which emphasizes *mianzi*, social etiquette, and politeness (Tsui and Farh, 1997). Hence, Chinese employees are more likely to endure when suffering tease or disparagement from their supervisors. Nevertheless, perceptual response patterns to workplace events are associated with cultural context (Levine et al., 2011). Accordingly, we encourage future research to replicate these results in other cultures or countries to improve the generalizability of the findings.

## Data availability statement

The raw data supporting the conclusions of this article will be made available by the authors, without undue reservation.

## Ethics statement

The studies involving human participants were reviewed and approved by Ethical Review Board of Beijing Institute of Petrochemical Technology. The patients/participants provided their written informed consent to participate in this study.

## Author contributions

DL made substantial contributions to the supervision, acquisition and analysis. PL made contributions to the conception of the work and drafting the work. SL made contributions to interpretation of data, JH revised the manuscript critically for important intellectual content. All authors contributed to the article and approved the submitted version.

## Conflict of interest

The authors declare that the research was conducted in the absence of any commercial or financial relationships that could be construed as a potential conflict of interest.

## Publisher’s note

All claims expressed in this article are solely those of the authors and do not necessarily represent those of their affiliated organizations, or those of the publisher, the editors and the reviewers. Any product that may be evaluated in this article, or claim that may be made by its manufacturer, is not guaranteed or endorsed by the publisher.
